# TRIM29 Reverses Oxaliplatin Resistance of P53 Mutant Colon Cancer Cell

**DOI:** 10.1155/2021/8870907

**Published:** 2021-03-22

**Authors:** Guoqiong Lei, Sushun Liu, Xin Yang, Chao He

**Affiliations:** ^1^Department of Neurosurgery, The Second People's Hospital of Hunan Province, Changsha, Hunan 410007, China; ^2^Department of General Surgery, The Second Xiangya Hospital, Central South University, Changsha, Hunan 410011, China

## Abstract

**Background:**

Oxaliplatin is the first-choice chemotherapy method for patients with advanced colon cancer. However, its resistance leads to treatment failure for many patients. In our experiments, we aim to elucidate the associations among TRIM29 protein, mutant P53, and the resistance of colon cancer cells to oxaliplatin.

**Methods:**

HCT116 and HT-29 cells were cultured and transfected with plasmids pIRES2-ZsGreen1-TRIM29-flag. Western blot and real-time qRT-PCR were utilized to examine the protein and mRNA expressions of TRIM29 and other related markers, respectively. MTT assay was utilized to determine the cell growth rate and generate the inhibition curve. Continuous culture in low-concentration oxaliplatin was conducted to construct oxaliplatin-resistant cell lines. The coimmunoprecipitation method and immunofluorescence detection were used to examine the interaction between TRIM29 and mutant P53 protein in HT29 cells.

**Results:**

We successfully transfected pIRES2-ZsGreen1-TRIM29-flag into HCT116 and HT29 cells, which were utilized in the whole experiments. TRIM29 significantly increased the sensitivity of P53 mutant colon cancer cell HT29 to oxaliplatin. The oxaliplatin-resistant model of P53 mutant colon cancer cell HT29 was successfully constructed. TRIM29 physically bound with mutant P53 and retained it in the cytoplasm from the nucleus, which inhibited its transcription function of downstream genes such as MDR1. In addition, TRIM29 successfully reversed the resistance of HT29-OX resistant cell model to oxaliplatin.

**Conclusion:**

In mutant P53 colon cancer cell HT29, TRIM29 greatly increased the sensitivity of HT29 to oxaliplatin and reverse oxaliplatin resistance. The underlying mechanism is TRIM29 may increase the sensitivity of HT29 to oxaliplatin by blocking the transcriptional function of mutant P53, which inhibits the transcription function of its downstream gene such as MDR1.

## 1. Introduction

In recent years, the population of colorectal cancer patients has gradually increased [1]. Most patients suffer from severe symptoms such as intestinal obstruction and bleeding at the time of diagnosis, resulting in remote metastasis and late tumor stages (stages III and IV) [2]. Systemic chemotherapy is recommended for unresectable patients before or after surgery to reduce tumor recurrence and metastasis, as well as improving the five-year survival rate [2, 3]. Oxaliplatin is the first-choice chemotherapy method for patients with high-risk relapses and lymph node metastasis [4, 5]. It acts on DNA by generating hydration derivatives to form intrachain and interchain cross-links [6]. The formation of DNA conjugates can initiate the DNA damage response of cells, which induces apoptosis to achieve its antitumor effect [7, 8].

Due to the resistance development to chemotherapy drugs in tumor cells, many tumor patients inevitably relapse and metastasize after several effective chemotherapies [9, 10]. Such chemotherapy resistance places great hurdles to colon cancer patients from recovery or survival. Among colon cancer patients, more than 60% of them have P53 mutations [11–13]. It is widely believed that P53 mutation indicates poor clinical staging, prognosis, and resistance to colon cancer [14–16]. P53-deficient cells are highly sensitive to DNA-damaging drugs, while P53 mutant cells are highly resistant to 5-FU [17]. Clinical studies also suggest that mutant P53 increases the resistance of colorectal cancer patients to chemotherapy [15, 18, 19]. P53 mutation is one of the important factors for colon cancer chemotherapy resistance, which drives us to explore more effective methods to improve therapy outcomes.

TRIM29 gene mainly exists in the cytoplasm of the cell and binds to a variety of cytoskeletal proteins. It is found that TRIM29 can bind to P53, which could negatively regulate the nuclear transcription of P53 and block its functions [20]. Besides, TRIM29 enhances tumor growth and metastasis in vivo, is highly expressed in many tumors, and could promote tumor growth, such as colon cancer and prostate cancer [21–23]. It is generally considered to be a cancer-promoting factor. However, TRIM29 is downregulated in other tumors such as breast cancer and prostate cancer [23–26]. As TRIM29 acts as a double-edged sword for tumor growth, it remains to exert important effects in both the occurrence and the development of tumors.

Previous researchers established that TRIM29 showed tumor-suppressive effects in P53 mutant Saos-2 cells [27] and BT-549 cells [27] and tumor-promoting effects in P53 wild-type colon cancer cell lines RKO [22] and HEK93 [21]. TRIM29 is associated with both wild-type P53 and mutant P53. We speculate that its dual function may be related to the P53 status of tumor cells. Therefore, we aim to elucidate the associations among TRIM29 protein, mutant P53, and the resistance of colon cancer cells to oxaliplatin in the experiments.

## 2. Materials and Methods

### 2.1. Cell Culture

HCT116 and HT-29 cells (Cellbank, Shanghai Academy of Life Sciences, Chinese Academy of Sciences) were cultured in 1640 medium containing 10% inactivated fetal bovine serum (Thermo Fisher Scientific, Shanghai, China) in an incubator at 37°C with humidity and 5% CO_2_. HCT116 cells in the logarithmic growth phase were seeded in 96-well culture plates with 2 × 10^3^ cells per well in 200 *μ*L volume. After adhered for 24 h, the plasmids pIRES2-ZsGreen1-TRIM29-flag (HG-HO101913, HonorGene) and pIRES2-ZsGreen1 (#632478, Clontech) were transfected into HCT116 cells. HT29 cells were seeded in 96-well culture plates with 2 × 10^3^ cells per well in 200 *μ*L volume. After adhered for 24 h, the plasmids pIRES2-ZsGreen1-TRIM29-flag and pIRES2-ZsGreen1 were transfect into the HT29 cells.

### 2.2. Cell Transfection

pCDNA3.1-TRIM29-flag and pCMV-HA-p53-R273H were purchased from HonorGene (Changsha, China). According to different treatment methods, the experimental cells are divided into (1) HT29 group: HT29 cells in the natural state; (2) HT29-NC group: HT29 cells transfected with pIRES2-ZsGreen1 plasmid; (3) HT29-TRIM29 group: transfected pIRES2- HT29 cells of ZsGreen1-TRIM29 plasmid; (4) HCT116 group: HCT116 cells in the natural state; (5) HCT116-NC group: HCT116 cells transfected with pIRES2-ZsGreen1 plasmid; and (6) HCT116-TRIM29 group: transfected pIRES2-ZsGreen1- HIM116 cells with TRIM29 plasmid. The cells (HT29 or HCT116) had transfection with plasmid pIRES2-ZsGreen1 (HT29-NC or HCT116-NC) or pIRES2-ZsGreen1-TRIM29-flag (HT29-TRIM29 or HCT116-TRIM29) according to the above groups.

### 2.3. Western Blot

HT29 cells were lysed and centrifuged. The protein content was measured using the BCA kit (Beyotime Institute of Biotechnology, Jiangsu, China), and the concentration was calculated. SDS-PAGE electrophoresis was performed and the proteins were transferred to the nitrocellulose membrane. The membrane was then stained with a 2% Ponceau dye solution. After washing, the membrane was blocked. Then, primary antibodies TRIM29 (ab108627, 1 : 5000, Abcam), MDR1 (ab170904, 1 : 2000, Abcam), P53 (60283-2-Ig, 1 : 5000, Proteintech), HA (ab1424, 1 : 7000, Abcam), Flag (ab205606, 1 : 10,000, Abcam), and *β*-actin (66009-1-Ig, 1 : 5000, Proteintech) were added, with an incubation time of 2–4 hours at room temperature. After washing, a horseradish peroxidase-labeled secondary antibody HRP-goat anti-mouse IgG (SA00001-1, 1 : 5000, Proteintech) or HRP-goat anti-rabbit IgG (SA00001-2, 1 : 6000, Proteintech) was added, with an incubation time of 1 hour at room temperature. ECL chemiluminescence was utilized for visualization.

### 2.4. Real-Time Quantitative Reverse Transcription PCR (Real-Time qRT-PCR)

Trizol was utilized to extract total RNA from target cells. Using the cDNA as a template, the target gene and the internal reference GAPDH fragment were amplified, respectively. Real-time PCR was used to quantify the mRNA level of the target gene in each group of cells. The primers utilized in the experiments were as follows:

  P53-f: 5′-CCACCATCCACTACAACTACAT-3′
  P53-r: 5′-CCCAGGACAGGCACAAAC-3′
  MDR1-f: 5′-CAACGGAAGCCAGAACAT-3′
  MDR1-r: 5′-AATCAGCCTCACCACAGA-3′
  TRIM29-f: 5′-GCATAGCATCAGCGACTC-3′
  TRIM29-r: 5′-GTTCCTCTCAATGAAGTTACG-3′
  GAPDH-f: 5′-TGCACCACCAACTGCTTAGC-3′
  GAPDH-r: 5′-GGCATGGACTGTGGTCATGAG-3′

### 2.5. MTT Experiment Steps

20 *μ*l MTT solution (5 mg/ml) per well was added to cells from each group, with an incubation period of 4 h in a 37°C, 5% CO_2_ incubator. The culture supernatant in the well was carefully aspirated to avoid aspirating purple crystals. 150 *μ*l DMSO was added to each well, which is under shaking for 10 minutes to fully dissolve the crystals. The light absorption at 490 nm was utilized to measure the results. The cell growth curve was plotted with time as abscissa and absorbance as ordinate.

### 2.6. Determination of the Inhibition Curve

After incubating at 37°C in a 5% CO_2_ incubator for 48 hours, oxaliplatin was added in sequence to make the final concentration of 0 *μ*mol/L, 25 *μ*mol/L, 50 *μ*mol/L, 100 *μ*mol/L, 200 *μ*mol/L, and 400 *μ*mol/L. After 48 hours of cultivation, MTT was used for the detection of OD value. The inhibition rate of each group equals 1-OD value treated by oxaliplatin (25 *μ*mol/L, 50 *μ*mol/L, 100 **μ**mol/L, 200 **μ**mol/L, and 400 *μ*mol/L)/OD value without oxaliplatin (0 *μ*mol/L). The inhibition curve was drawn with oxaliplatin concentration on the abscissa and the inhibition rate on the ordinate.

### 2.7. Coimmunoprecipitation Method

The coimmunoprecipitation method was used to examine the interaction between TRIM29 and mutant P53 protein in HT29 cells. Briefly, 800 *μ*l IP lysis solution was first added to the cell pellet and lyse for 30 minutes. It was centrifuged at 12,000 rpm for 15 minutes at 4°C. The centrifuged supernatant was transferred to a new 1.5 ml centrifuge tube, added with 2 *μ*g normal mouse lgG (B900620, Proteintech), 2 *μ*g normal rabbit lgG (B900610, Proteintech), mouse-derived P53 antibody, and rabbit-derived TRIM29 antibody. The mixture was incubated overnight at 4°C. 20 *μ*l protein A/G agarose beads were added and mixed with 200 *μ*l IP lysate. The cell lysate was added and incubated with antibody overnight to the pretreated protein A/G agarose beads. After coimmunoprecipitation, the mixture was centrifuged at 3000 rpm for 3 min at 4°C. The supernatant was carefully removed, and placed in a new 1.5 ml centrifuge tube. The agarose beads were washed with 400 *μ*l IP lysis solution 4 times. The precipitate was collected. The supernatant was retained.

### 2.8. Immunofluorescence Detection

Immunofluorescence detection was performed to explore the interaction between TRIM29 and mutant P53 protein in HT29 cells transfected with pCDNA3.1-TRIM29-flag or pCMV-HA-p53-R273H. The cells were divided into 3 groups: (1) NC group: HT-29 cells cotransfected with pCDNA3.1(+) and pCMV-HA plasmids; (2) p53 group: HT-29 cells cotransfected with pCDNA3.1(+) and pCMV-HA-p53-R273H plasmid; and (3) 53+TRIM29 group: HT-29 cells cotransfected with pCDNA3.1-TRIM29-flag and pCMV-HA-p53-R273H plasmids. After 48 hours of transfection, immunofluorescence detection was performed. Briefly, the cells were cultured in DMEM medium containing 10% inactivated fetal bovine serum in an incubator containing 37% saturated humidity and 5% CO_2_. After fixation with 4% paraformaldehyde, 0.4% Triton 100 was permeated through the membrane for 15 minutes. The primary antibody (rabbit anti-HA antibody, mouse anti-Flag antibody) was added and incubated at 4 degrees overnight. On the third day, the saline was washed twice. Secondary antibodies labeled with FITC and TRITC (1 : 50, TRITC-anti-mouse; FITC-anti-rabbit, KPL) were added and incubated at room temperature for 2 h. Pictures were taken using a laser confocal microscope.

### 2.9. Statistical Methods

SPSS 19.0 was utilized to perform an independent sample *t*-test. The calculation of IC50 was performed using SPSS19 regression analysis. Drug resistance index = IC50 of drug-resistant cells/IC50 of parental cells. Data among multiple groups were compared using one-way analysis of variance (ANOVA) followed by Tukey's post hoc test. Data among multiple groups in skew distribution were tested by the nonparametric Kruskal–Wallis *H* test. *P* less than 0.05 was considered statistically significant.

## 3. Results

### 3.1. pIRES2-ZsGreen1-TRIM29-Flag Was Successfully Transfected

**Figure 1:**
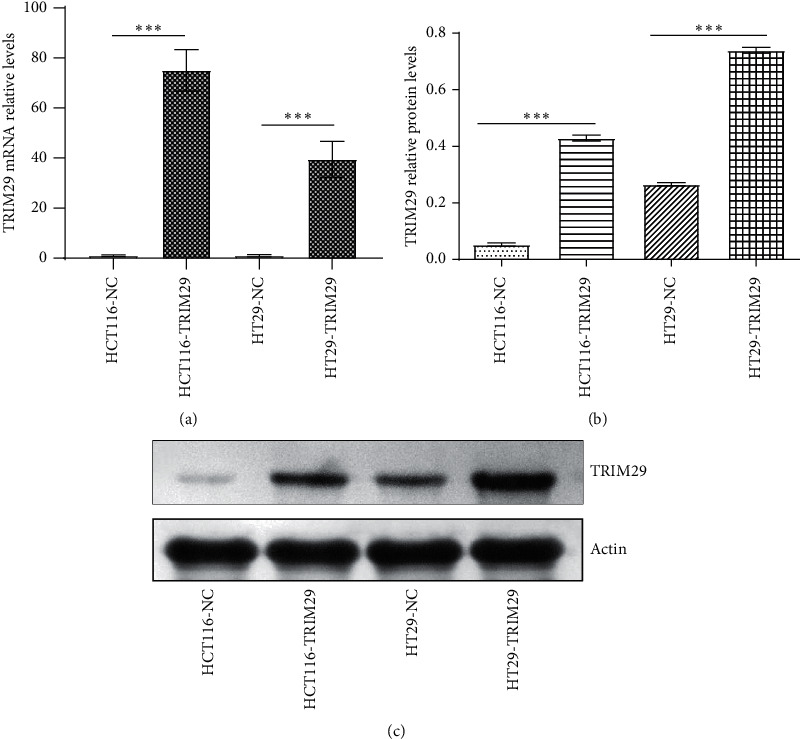
pIRES2-ZsGreen1-TRIM29-flag was successfully transfected into HCT116 and HT29 cells. (a). After transfection with the pIRES2-ZsGreen1-TRIM29-flag plasmid, the mRNA expression of TRIM29 in HCT116 and HT29 cells increased. (b). After transfection with pIRES2-ZsGreen1-TRIM29-flag plasmid, the protein expression of TRIM29 in HCT116 and HT29 cells increased. (c). The Western blot band image of protein expression of TRIM29 in HCT116 and HT29 cells. ^*∗∗∗*^*P* < 0.05. Comparisons among multiple groups were analyzed using one-way ANOVA.

Figure 1 demonstrated that we successfully transfected pIRES2-ZsGreen1-TRIM29-flag into HCT116 and HT29 cells.  Figure 1(a) shows that the mRNA expression of TRIM29 in HCT116 and HT29 cells increased significantly after the transfection of the pIRES2-ZsGreen1-TRIM29-flag plasmid. Figures 1(b) and 1(c) illustrated that TRIM29 protein expression in HCT116 and HT29 cells increased after transfection with the pIRES2-ZsGreen1-TRIM29-flag plasmid. The above results indicated that we successfully transfected pIRES2-ZsGreen1-TRIM29-flag into HCT116 and HT29 cells, which could be utilized in the following experiments.

### 3.2. TRIM29 Significantly Increased the Sensitivity of P53 Mutant Colon Cancer Cell HT29 to Oxaliplatin

**Figure 2:**
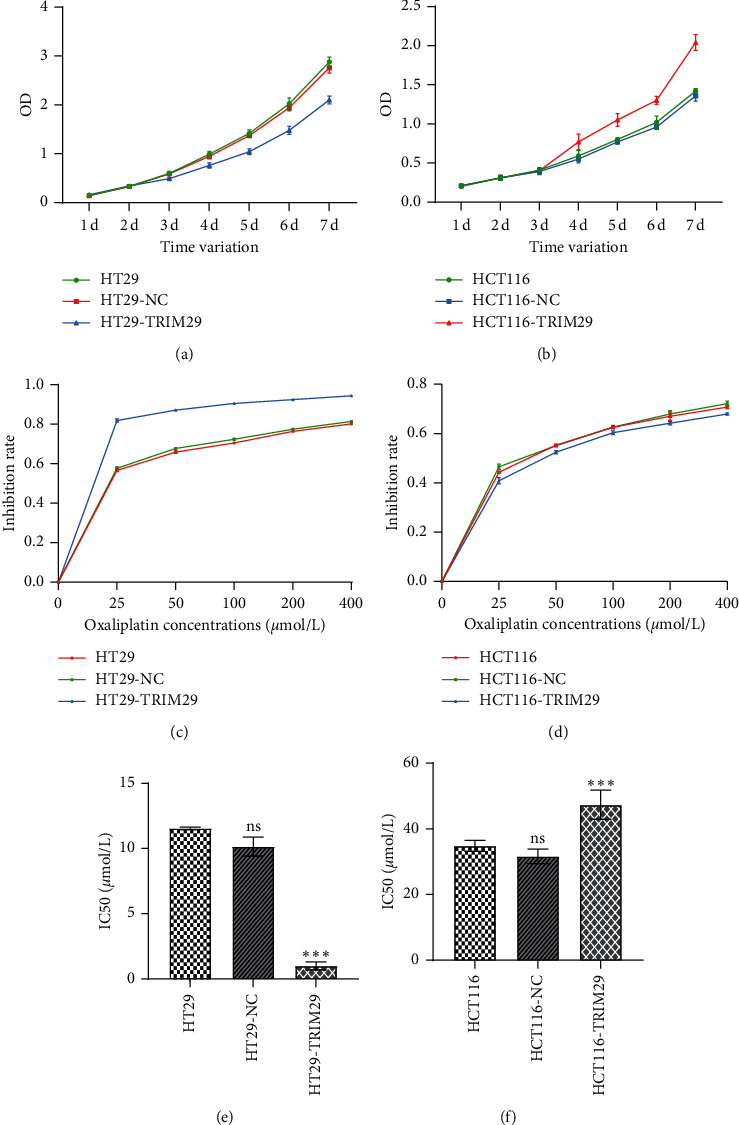
TRIM29 significantly increased the sensitivity of P53 mutant colon cancer cell HT29 to oxaliplatin. (a) HT29 cell growth curve after transfection of pIRES2-ZsGreen1-TRIM29-flag plasmid. (b) HCT116 cell growth curve after transfection of pIRES2-ZsGreen1-TRIM29-flag plasmid. (c) HT29 cell inhibition curve to oxaliplatin after transfection of pIRES2-ZsGreen1-TRIM29-flag plasmid. (d) HCT116 cell inhibition curve to oxaliplatin after transfection of pIRES2-ZsGreen1-TRIM29-flag plasmid. (e) IC50 change of HT29 cells after transfection of pIRES2-ZsGreen1-TRIM29-flag plasmid. (f) IC50 change of HCT116 cells after transfection of pIRES2-ZsGreen1-TRIM29-flag plasmid. ns: no significant; ^*∗∗∗*^*P* < 0.05. Comparisons among multiple groups were analyzed by one-way ANOVA, followed by Tukey's post hoc test. Data among multiple groups in skew distribution were tested by the nonparametric Kruskal–Wallis *H* test.

Figures 2(a) and 2(b) show the cell growth curve after the transfection of pIRES2-ZsGreen1-TRIM29-flag plasmid. HCT116 grew significantly faster from the fourth day. On the seventh day, the number of cells in the transfection group was about 1.4 times higher than the number of cells in the parent group. Figures 2(c) and 2(d) show the inhibition curves of different concentrations of oxaliplatin treatments on HT29 and HCT116 groups. It was obvious that TRIM29 elevated the inhibition rate of HT29 to oxaliplatin, while it made little difference in HCT116 cells. Figures 2(e) and 2(f) demonstrated the IC50 changes among different groups. After transfection of pIRES2-ZsGreen1-TRIM29-flag plasmid, IC50 of oxaliplatin of HCT116 increased from 34.89 *μ*mol/L to 47.26 *μ*mol/L. The drug resistance index was 1.35. The sensitivity of HCT116 to oxaliplatin was slightly reduced. For HT29, oxaliplatin IC50 decreased from 11.54 *μ*mol/L to 0.98 *μ*mol/L, and the drug resistance index was 0.08. The sensitivity of HT29 to oxaliplatin was significantly improved. In summary, TRIM29 significantly increased the sensitivity of P53 mutant colon cancer cell HT29 to oxaliplatin.

### 3.3. The Oxaliplatin-Resistant Model of P53 Mutant Colon Cancer Cell HT29 Was Successfully Constructed

**Figure 3:**
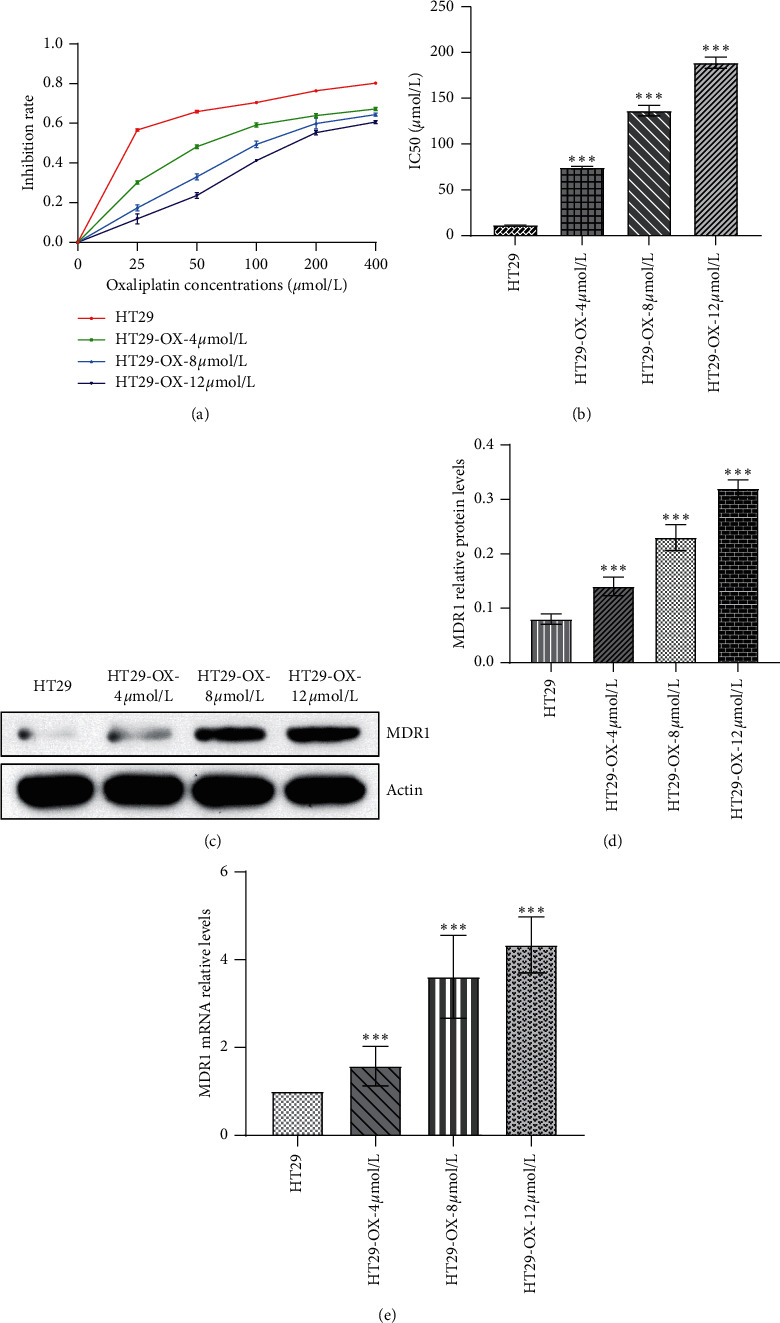
The oxaliplatin-resistant model of P53 mutant colon cancer cell HT29 was successfully constructed. (a) Inhibitory curves of HT29, HT29-OX-4 *μ*mol/L, HT29-OX-8 *μ*mol/L, and HT29-OX-12 *μ*mol/L. (b) The IC50 value of HT29, HT29-OX-4 *μ*mol/L, HT29-OX-8 *μ*mol/L, and HT29-OX-12 *μ*mol/L. (c) Relative protein expression of MDR1 in HT29, HT29-OX-4 *μ*mol/L, HT29-OX-8 *μ*mol/L, and HT29-OX-12 *μ*mol/L. (d) The quantitative analysis of (c). (e) Relative mRNA level of MDR1 in HT29, HT29-OX-4 *μ*mol/L, HT29-OX-8 *μ*mol/L, and HT29-OX-12 *μ*mol/L. ^*∗∗∗*^*P* < 0.05. Data among multiple groups were analyzed by one-way ANOVA, followed by Tukey's post hoc test. Data among multiple groups in skew distribution were tested by the nonparametric Kruskal–Wallis *H* test.

Next, we successfully constructed P53 mutant colon cancer cell HT29-oxaliplatin-resistant model.  Figure 3(a) shows the inhibition curves of HT29, HT29-4 *μ*mol/L oxaliplatin, HT29-8 *μ*mol/L oxaliplatin, and HT29-12 *μ*mol/L oxaliplatin. With higher concentrations of oxaliplatin, HT29 cells showed better inhibition effects.  Figure 3(b) illustrates the IC50 of the parental and three drug-resistant cell lines. As the concentration of drug resistance increased, the IC50 of drug-resistant cells increased accordingly. The IC50 of the parental and three drug-resistant cell lines were 11.5 ± 0.1 *μ*mol/L, 74.4 ± 1.3 *μ*mol/L, 136.5 ± 5.8 *μ*mol/L, and 188.9 ± 6.2 *μ*mol/L, respectively. Figures 3(c)–3(e) demonstrate that the expression of MDR1 in three drug-resistant cells gradually increased with the increase of drug resistance concentration. The mRNA level of HT29-OX-12 *μ*mol/L increased to 4.3 times of the parent (Figure 3(e)), and the protein level increased to 2 times of the parent (Figures 3(c) and 3(d)). These results indicated our successful construction of the oxaliplatin-resistant model of P53 mutant colon cancer cell HT29.

### 3.4. TRIM29 Physically Binds with Mutant P53 to Prevent the Mutant p53 to Transfer from the Cytoplasm to the Nucleus and the Transcription

**Figure 4:**
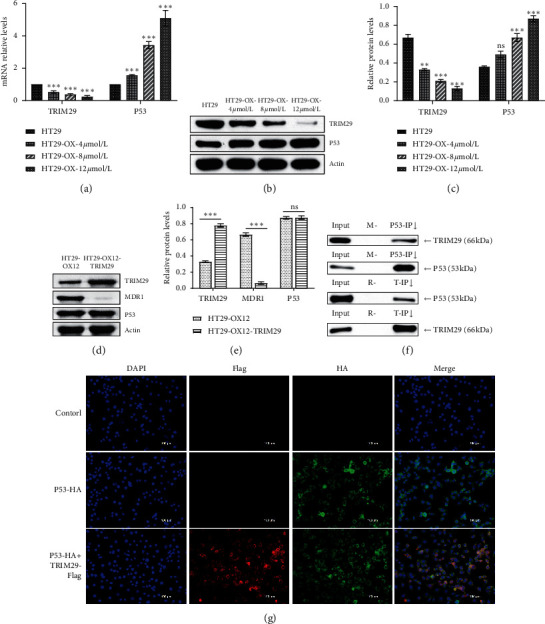
TRIM29 physically binds with mutant P53. (a) qRT-PCR was used to detect the expression of TRIM29 and P53 in the three drug-resistant cell models with the increase of drug resistance concentration. After the HT29-OX-12 *μ*mol/L cells were transfected with pIRES2-ZsGreen1-TRIM29-flag plasmid, the changes of P53 and MDR1 expression were detected. (b) The Western blot method was used to detect the expression of TRIM29 and P53 in the three drug-resistant cell models. (c) The quantitative analysis of (b). (d) After the HT29-OX-12 *μ*mol/L cells were transfected with pIRES2-ZsGreen1-TRIM29-flag plasmid, the protein expression changes of P53 and MDR1 expression were detected. (e) Quantitative analysis of (d). (f) Coimmunoprecipitation method was used to detect the interaction between TRIM29 and mutant P53 protein in HT29 cells. (g) Immunofluorescence detection of the interaction between TRIM29 and mutant P53 protein in HT29 cells. Scale bar = 100 *μ*m. The magnification is 400 times; ns: not significant; ^*∗∗*^*P* < 0.001; ^*∗∗∗*^*P* < 0.001. The unpaired *t*-test was used to analyze comparisons between two groups, and comparisons among multiple groups were analyzed by one-way ANOVA, followed by Tukey's post hoc test.

It was found that TRIM29 can bind to wild-type p53 and does not directly regulate the transcription of p53 [28]. TRIM29 is a cytoplasmic protein, which enables p53 protein to be exported from the nucleus, thereby inhibiting the function of p53 to regulate the transcription of downstream target genes [20]. As shown in Supplementary Figure 1, TRIM29 binds to amino acids 320–393 of the p53 tetramerization domain (TET), and p53 binds to amino acids 1–200 of TRIM29. We detected the changes in the expression of P53 and TRIM29 in HT29 with resistance to different concentrations of oxaliplatin after the transfection of the pIRES2-ZsGreen1-TRIM29-flag plasmid. Figures 4(a)–4(c) show the relative mRNA levels and protein expression of TRIM29 and P53, respectively. With the increase in the resistance to different concentrations of oxaliplatin, the expression of TRIM29 gradually decreased and the expression of P53 gradually increased. After the HT29-OX-12 *μ*mol/L cells were transfected with the pIRES2-ZsGreen1-TRIM29-flag plasmid, the changes of P53 and MDR1 expression were detected (Figures 4(d) and 4(e)). After the transfection of TRIM29 plasmid, the expression of TRIM29 increased, there was no significant change in the expression of drug-resistant strain P53, and the expression of MDR1 decreased. The above results suggest that TRIM29 may prevent mutant p53 downstream gene transcription.

Then, we aim to explore the inner mechanism of TRIM29 in the reversed resistance of HT29 to oxaliplatin.  Figure 4(f) shows the method of immunoprecipitation to detect the interaction between TRIM29 and mutant P53 protein in HT29 cells. What is more, we detected Flag tag and HA tag expression in pCDNA3.1-TRIM29-flag and pCMV-HA-p53-R273H plasmid transfected HT29 and HCT116 cells by Western blot. As shown in Supplementary Figures 2 and 3, the results showed that pCDNA3.1-TRIM29-flag and pCMV-HA-p53-R273H were successfully transfected. After transfection with pCDNA3.1-TRIM29-flag plasmid, the protein expression of Flag in HCT116 and HT29 cells increased. After transfection with pCMV-HA-p53-R273H plasmid, the protein expression of HA in HCT116 and HT29 cells also increased. Then, Figure 4(g) demonstrates the immunofluorescence detection of the interaction between TRIM29 and mutant P53 protein in HT29 cells. In P53 mutant colon cancer cell HT29, TRIM29 and mutant P53 protein bind with each other. At least a portion of the p53 mutant enters the nucleus from the cytoplasm, thereby eliminating the mutant p53 transcriptional function and changing drug resistance.

### 3.5. TRIM29 Successfully Reversed the Resistance of HT29-OX Resistant Cell Model to Oxaliplatin

**Figure 5:**
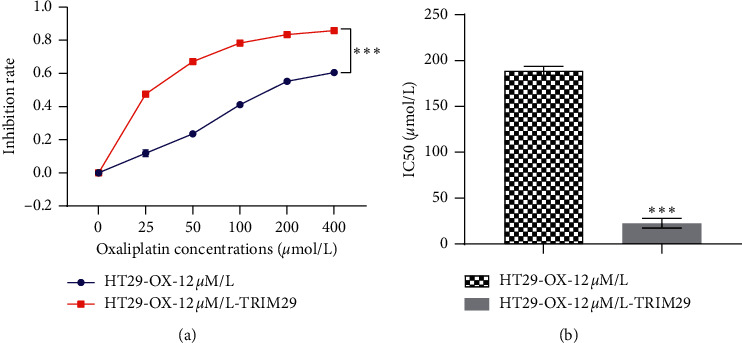
TRIM29 successfully reversed the resistance of the HT29-OX resistant cell model to oxaliplatin. (a) Inhibition curve in HT29-oxaliplatin-12 *μ*mol/L cells before and after transfection of pIRES2-ZsGreen1-TRIM29 plasmid. (b) Change of oxaliplatin IC50 in HT29-OX-12 *μ*mol/L cells before and after transfection of pIRES2-ZsGreen1-TRIM29 plasmid. Compared with HT29-12 *μ*mol/L oxaliplatin group, ^*∗∗∗*^*P* < 0.05. The unpaired *t*-test was used to analyze comparisons between two groups. Data among multiple groups in skew distribution were tested by the nonparametric Kruskal–Wallis *H* test.

Figure 5(a) shows the inhibition curve to oxaliplatin in HT29-OX-12 *μ*mol/L cells before and after transfection with pIRES2-ZsGreen1-TRIM29-flag plasmid by MTT method.  Figure 5(b) demonstrates the changes of IC50 in HT29-OX-12 *μ*mol/L cells before and after transfection of pIRES2-ZsGreen1-TRIM29-flag plasmid. After transfection with TRIM29, the drug-resistant strain HT29-12 *μ*mol/L oxaliplatin greatly increased the sensitivity to oxaliplatin, and the IC50 decreased from 188.9 ± 6.2 *μ*mol/L to 22.6 ± 6.7 *μ*mol/L. From the above results, we could conclude that TRIM29 successfully reversed the resistance of HT29-OX resistant cell model to oxaliplatin.

## 4. Discussion

The TRIM protein family consists of three characteristic zinc-binding domains, including a ring structure, a type 1 B-box, and a type 2 B-box structure, followed by a coiled-coil region [29–31]. Although the biological function of the TRIM protein domain has not been fully elucidated, some TRIM proteins play important roles in viral replication, signaling, development, and human diseases, especially tumors [32]. However, there is no evidence that TRIM29 can directly inhibit or activate transcription. Previous studies found that TRIM29 is highly correlated with P53 [20]. In our study, we found that the TRIM29 cytoplasmic protein could bind to mutant p53, which helps prevent the mutant p53 to transfer from the cytoplasm to the nucleus. Such physical binding could block mutant p53's role in nuclear transcription and decrease the expression of downstream genes such as MDR1, thus resulting in oxaliplatin resistance. Our findings are consistent with former studies in the interaction between TRIM29 and wild-type p53 and provide new insight into the p53-related oxaliplatin resistance.

In this study, we chose HT29 and HCT116 as our colon cancer cell lines. HT29 and HCT116 colon cancer cell lines are two kinds of cells with different degrees of assimilation. HT29 is moderately differentiated and can be induced to differentiate into intestinal epithelial cells, while HCT116 is a highly invasive colon cancer cell line in an undifferentiated state [33–35]. HT29 cells are human intestinal epithelial cells which produce the secretory component of immunoglobulin A (IgA) and carcinoembryonic antigen (CEA). HT29 cells are used for tumourigenicity studies [36, 37]. HCT116 cells have been widely used in the study of biological characteristics of malignant tumor cells, the mechanism of anticancer drugs, and the screening of anticancer drugs. Recent studies have shown that most of the HCT116 cells have the characteristics of tumor stem cells and can be used as the ideal research object of tumor stem cells [38, 39].

TRIM29 promotes aggregation in *β*-catenin cells through the *β*-catenin/TCF pathway [21]. TRIM29 is highly expressed in many tumors and promotes tumor growth [21–23]. However, the expression of TRIM29 is suppressed in some tumors [24, 40–42]. For example, TRIM29 is sometimes overexpressed and sometimes downregulated especially in prostate cancer. In breast cancer, TRIM29 also exhibits tumor-suppressive effects [25], and the inhibition of TRIM29 expression is associated with certain tumor malignant phenotypes [27]. In our experiments, we found that this dual function of TRIM29 may be related to the P53 status of tumor cells. In wild-type tumors of P53, TRIM29 appears to promote cancer development. In tumors of the p53 mutant type, TRIM29 shows cancer suppression effects. Our results showed that TRIM29 was slightly expressed in HT29, but not in HCT116 at the protein level. After transfection of the pIRES2-ZsGreen1-TRIM29-flag plasmid, the cells of both groups were highly expressed in mRNA and protein levels, suggesting that the plasmid was successfully transfected.

In P53 mutant colon cancer cell line HT29, TRIM29 inhibited the growth of HT29 and significantly increased the sensitivity of HT29 to oxaliplatin. TRIM29 may bind to not only wild-type P53 but also mutant P53 protein. Mutant P53 is closely related to tumor chemoresistance [43], especially in the chemical resistance of platinum drugs. Mutant P53 is involved in almost all currently known resistance mechanisms, such as promoting drug efflux [44], loss of DNA repair function [43], apoptosis resistance [45, 46], survival signal activation, and microenvironmental resistance [47–49]. From our experimental results, the sensitivity of HT29 colon cancer cells to oxaliplatin is greatly increased after the transfection of TRIM29, suggesting that TRIM29 may increase the sensitivity of HT29 to oxaliplatin by blocking the function of mutant P53. However, after TRIM29 transfection, the function of preventing wild-type P53 did not significantly increase the resistance of HCT116 to oxaliplatin, suggesting that oxaliplatin not just causes tumor apoptosis through P53 apoptosis pathway [50–52].

Wild-type p53 is a well-known tumor suppressor gene and a transcription factor, which promotes the expression of a series of tumor suppressor genes. The mutation of the p53 gene will lead to the loss of its tumor suppressor function and even obtain the function of promoting cancer as the gain of function. Based on our experiments, mutant p53 exerts its functions with dependence on the regulation of the expression of its downstream genes such as MDR1. The prediction of p53 status and expression of related molecules may help choose more appropriate chemotherapy for patients. Our research could enable precision medicine and individualized therapy for colon cancer patients. In future work, we will continue to investigate the potential working mechanism of mutant p53 and its major signaling pathways.

## 5. Conclusion

In colon cancer, TRIM29 showed promoting effect of tumor growth on cells expressing wild-type P53 (HCT116) and tumor-suppressive effect on the cells expressing mutant P53 (HT29). The dual effect of TRIM29 is related to p53 status. TRIM29 can bind both wild-type p53 and mutant p53, which prevents their nuclear transcription function. In mutant p53 colon cancer cells, TRIM29 prevents the transcriptional function of mutant p53, such as the downstream gene MDR1, which reverses the chemoresistance of mutant p53 colon cancer.

## Data Availability

All the data used to support the findings of this study are included within the article.

## Supplementary Materials

Supplementary Materials Supplementary Figure 1: the binding sites of TRIM29 and p53. Supplementary Figure 2: after transfection with pCDNA3.1-TRIM29-flag plasmid, Western blot detected the protein expression of Flag in HCT116 and HT29 cells. ^*∗∗∗*^*P* < 0.05. Supplementary Figure 3: after transfection with pCMV-HA-p53-R273H plasmid, Western blot detected the protein expression of HA in HCT116 and HT29 cells. ^*∗∗∗*^*P* < 0.05.
