# Supplementary figures and images for: TRIM29 Reverses Oxaliplatin Resistance of P53 Mutant Colon Cancer Cell
Source: Can J Gastroenterol Hepatol. 2021 Mar 22;2021:8870907. doi: 10.1155/2021/8870907 (PMC8007381; doi:10.1155/2021/8870907)

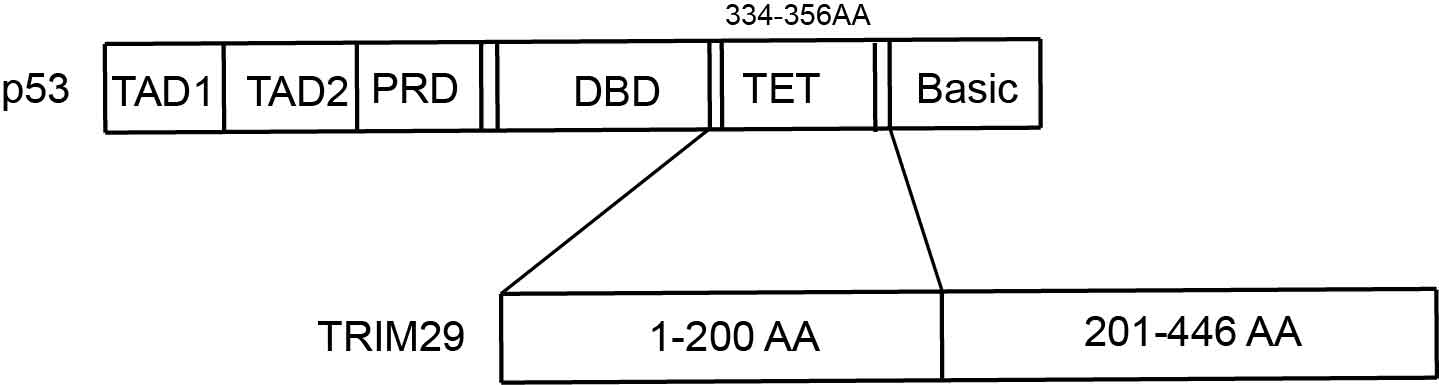

Supplement: Supplementary Materials — Supplementary Figure 1: the binding sites of TRIM29 and p53. Supplementary Figure 2: after transfection with pCDNA3.1-TRIM29-flag plasmid, Western blot detected the protein expression of Flag in HCT116 and HT29 cells. ∗∗∗P < 0.05. Supplementary Figure 3: after transfection with pCMV-HA-p53-R273H plasmid, Western blot detected the protein expression of HA in HCT116 and HT29 cells. ∗∗∗P < 0.05. [file 8870907.f1.zip › Supplementary Figure 1 (1).jpg]

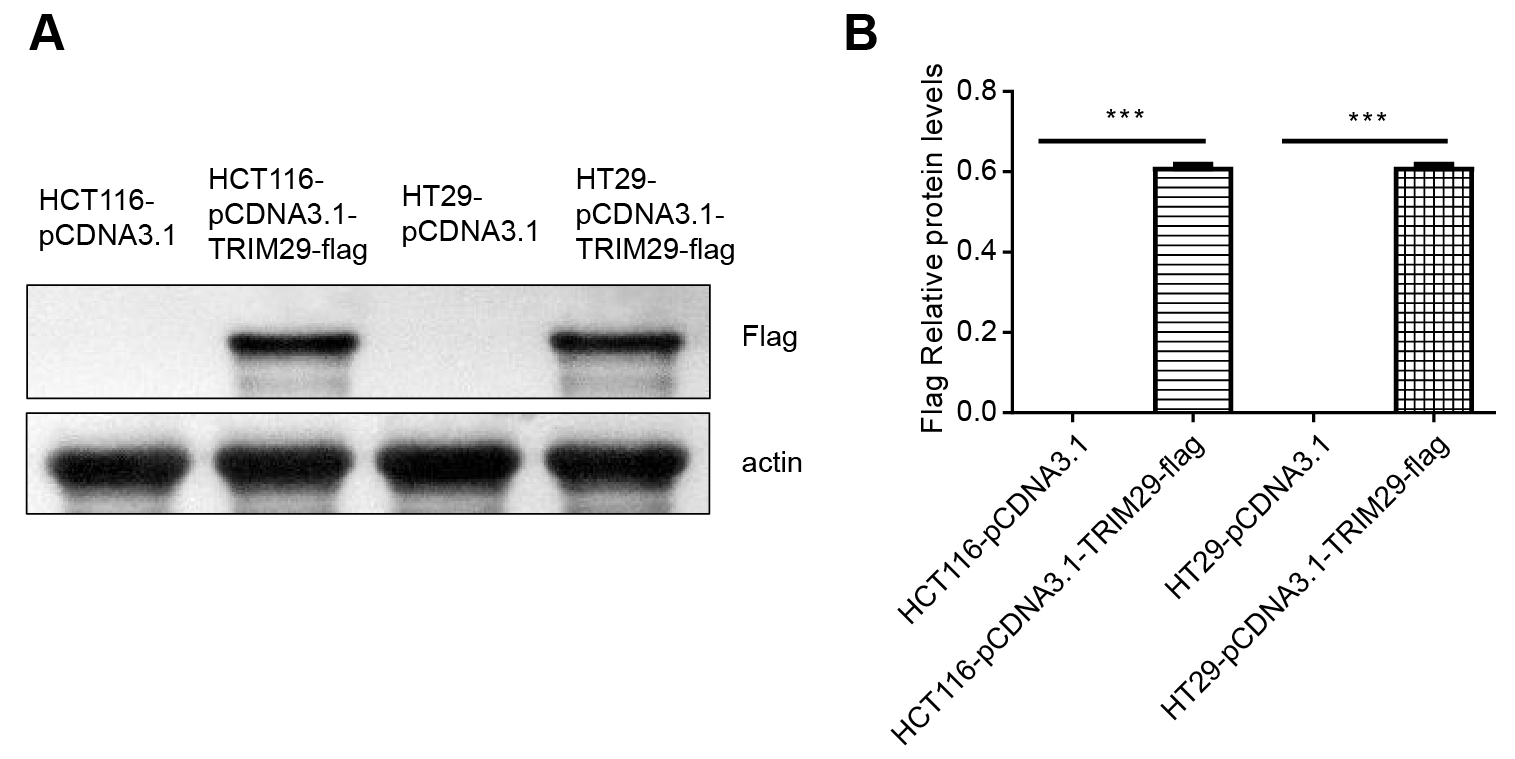

Supplement: Supplementary Materials — Supplementary Figure 1: the binding sites of TRIM29 and p53. Supplementary Figure 2: after transfection with pCDNA3.1-TRIM29-flag plasmid, Western blot detected the protein expression of Flag in HCT116 and HT29 cells. ∗∗∗P < 0.05. Supplementary Figure 3: after transfection with pCMV-HA-p53-R273H plasmid, Western blot detected the protein expression of HA in HCT116 and HT29 cells. ∗∗∗P < 0.05. [file 8870907.f1.zip › Supplementary Figure 2 (1).jpg]

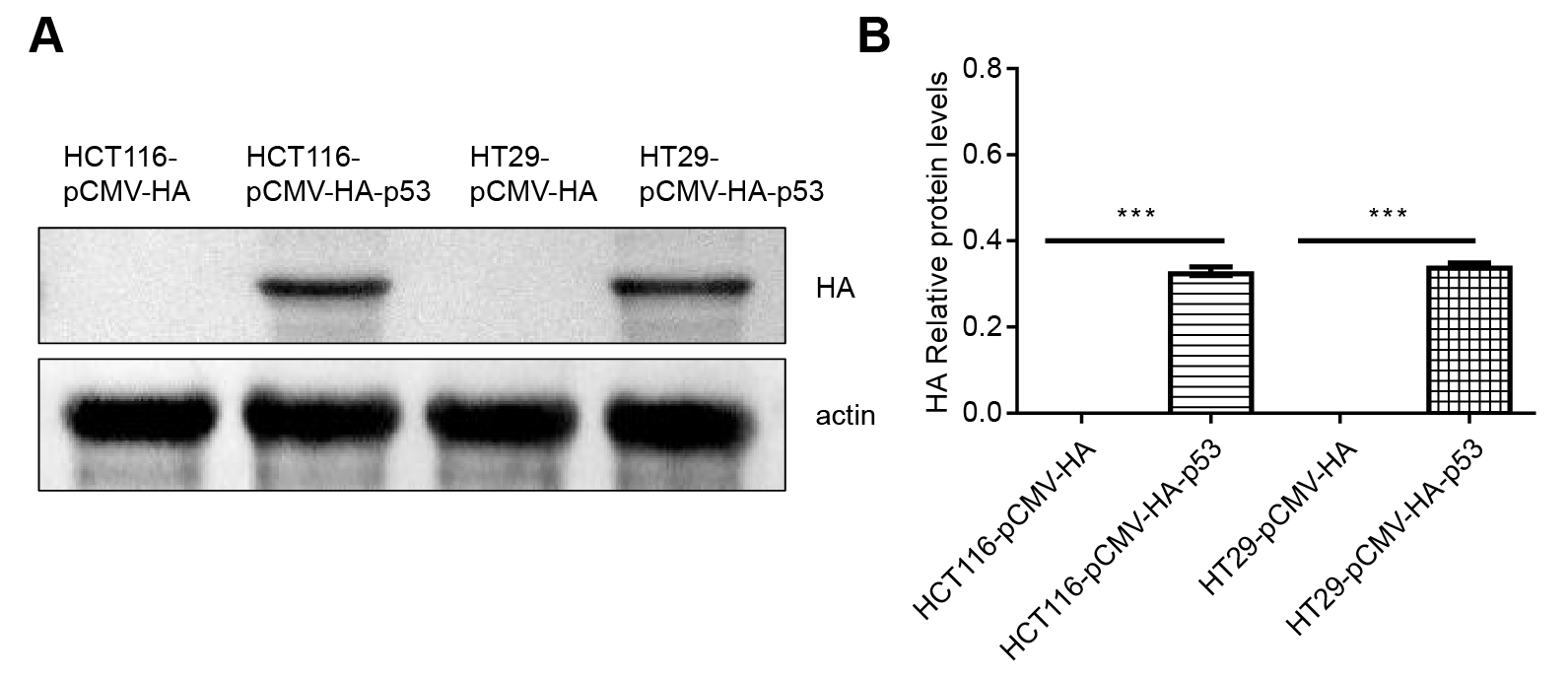

Supplement: Supplementary Materials — Supplementary Figure 1: the binding sites of TRIM29 and p53. Supplementary Figure 2: after transfection with pCDNA3.1-TRIM29-flag plasmid, Western blot detected the protein expression of Flag in HCT116 and HT29 cells. ∗∗∗P < 0.05. Supplementary Figure 3: after transfection with pCMV-HA-p53-R273H plasmid, Western blot detected the protein expression of HA in HCT116 and HT29 cells. ∗∗∗P < 0.05. [file 8870907.f1.zip › Supplementary Figure 3 (1).jpg]
